# Role of Ink4a/Arf Locus in Beta Cell Mass Expansion under Physiological and Pathological Conditions

**DOI:** 10.1155/2014/873679

**Published:** 2014-02-06

**Authors:** Elisabet Salas, Nabil Rabhi, Philippe Froguel, Jean-Sébastien Annicotte

**Affiliations:** ^1^European Genomic Institute for Diabetes (EGID), CNRS UMR 8199, Lille 2 University, 59000 Lille, France; ^2^Department of Genomics of Common Disease, Hammersmith Hospital, Imperial College London, London W12 0NN, UK

## Abstract

The ARF/INK4A (Cdkn2a) locus includes the linked tumour suppressor genes p16INK4a and p14ARF (p19ARF in mice) that trigger the antiproliferative activities of both RB and p53. With beta cell self-replication being the primary source for new beta cell generation in adult animals, the network by which beta cell replication could be increased to enhance beta cell mass and function is one of the approaches in diabetes research. In this review, we show a general view of the regulation points at transcriptional and posttranslational levels of Cdkn2a locus. We describe the molecular pathways and functions of Cdkn2a in beta cell cycle regulation. Given that aging reveals increased p16Ink4a levels in the pancreas that inhibit the proliferation of beta cells and decrease their ability to respond to injury, we show the state of the art about the role of this locus in beta cell senescence and diabetes development. Additionally, we focus on two approaches in beta cell regeneration strategies that rely on Cdkn2a locus negative regulation: long noncoding RNAs and betatrophin.

## 1. Introduction

Expansion and proliferation of insulin-secreting beta cells in pancreatic islets is a key highly regulated mechanism for establishing, maintaining, and adapting islet function to meet organism physiological demands. Understanding how the pieces of this mechanism fit together could improve development of islet replacement approaches; given that both type 1 and type 2 diabetes result from reduced beta cell mass and impaired beta cell functions. Islet beta cells expand in neonatal humans, mice, and other species, but this proliferation decays thereafter, which may promote pandemic (type 2) forms of diabetes mellitus [1]. Beta cell mass can be expanded by increasing beta cell replication, enlarging beta cell size, decreasing beta cell death, and promoting beta cell neogenesis. Murine beta cells proliferate slowly after birth, but this process can be accelerated under various conditions, including obesity, pregnancy, and stimulation by different beta cell mitogens, such as glucose, amino acids, insulin, prolactin, placental lactogen, and glucagon-like peptide-1. In addition to differentiation from pre-existing adult beta cell progenitors or transdifferentiation from another cell lineage, beta cell self-replication is considered to be the primary source for new beta cell generation in adult animals [2]. One of the approaches in diabetes research is investigating the function of beta cell cycle activators and inhibitors to elucidate the network by which beta cell replication could be increased to enhance beta cell mass and function.

**Figure 1:**
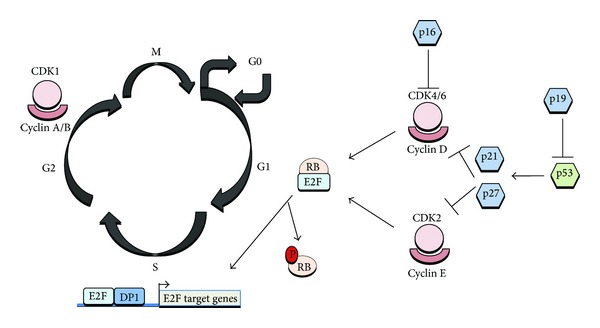
Mammalian cell cycle regulation. p16^Ink4b^ sequesters Cdk4 or Cdk6 inhibiting interactions with type D cyclins and preventing phosphorylation of pRB. Inactivation of CDK4/6 promotes Rb/E2F1 association triggering G1/S transition. Phosphorylation of pRB is essential for passage through the restriction point in G1. The cyclin D1-Cdk4 complex specifically phosphorylates the pRB protein leading to sequential phosphorylation by cyclin E-Cdk2 and release of free E2F. The phosphorylation of pRB, and relief of transcriptional inhibition by pRB induces S-phase entry. p53-dependent regulation by p21 and p27 contributes to checkpoint maintenance at later timepoints. Cdc2-Cyclin A/B binding contribute to phosphorylation of proteins involved in G2/M transition.

For the understanding of beta cell replication machinery it is important to focus on the cell cycle phases and checkpoints: the G1-S transition, S phase checkpoint, G2 to M transition and the Mitotic checkpoint (Figure 1). Checkpoint regulation mechanisms act through negative intracellular signals that arrest the cell cycle, rather than through the removal of positive signals that stimulate cell-cycle progression. Negative signals prevent cycle transition at the initiation of proliferation, replication and mitosis until the cellular conditions are the adequate for cell cycle progression. Sequential activation/inactivation of cyclin-dependent kinases (Cdks) is the primary means of cell cycle regulation. Cyclins confer substrate specificity and regulation to Cdk/cyclin complexes. According to that, in early G1 phase Cdk4 and/or Cdk6 are activated by D-type cyclins and initiate phosphorylation of the retinoblastoma protein (Rb) family. This triggers the release of E2F transcription factors and the subsequent activation and transcription of E2F responsive genes (including E- and A-type cyclins) required for cell-cycle progression. In the late G1 phase, Cdk2 binds to cyclin E and completes the phosphorylation of Rb, reinforcing the activation of E2F mediated transcription. These events lead to transition from the G1/S boundary point to S phase initiation.

Through S phase progression Cdk2 binds to cyclin A that contributes to DNA replication. During the G2/M transition, Cdk1/cyclin A complex is required for the initiation of prophase. Finally, Cdk1/cyclin B complexes are actively involved in the completion of mitosis. The negative regulation of Cdk/cyclin complexes relies on two families of Cdk inhibitors; the INK4 family (p16^INK4a^, p15^INK4b^, p18^INK4c^, p19^INK4d^) specifically bind to Cdk4 and Cdk6 and prevent D-type cyclin activity and the Cip/Kip family (p21^Cip1^/Waf1/Sdi1, p27^Kip1^, p57^Kip2^) inhibits Cdk2/cyclin E, Cdk2/cyclin A, Cdk1/cyclin A, as well as Cdk1/cyclin B activity (for review see [3]).

The p53 gene product is another key cell cycle check-point regulator at both the G1/S and G2/M and it has been shown to activate transcription of number of cell cycle genes and its essential role is to arrest cells in G1 after genotoxic damage allowing DNA repair prior to DNA replication and cell division. In response to massive DNA damage, p53 triggers the apoptotic cell death pathway [4].

Here we give an overview about the regulation points of ARF/INK4A locus transcription in beta cell, specifically in proliferation processes that give rise to cellular senescence and the development of diabetes. We state the more striking studies that shed a light in the approach of pancreatic beta cell proliferation and regeneration.

## 2. ARF/INK4A Locus Regulation in Beta Cells

Candidate gene and genome-wide association studies (GWAS) have identified several loci associated with type 2 diabetes and related traits. Specifically, genetic variants at CDKN2A/B locus have been associated with type 2 diabetes in many ethnic populations. A number of publications in the last five years confirm and validate that CDKN2A/B is a locus associated with genetic risk of type 2 diabetes development [5–8]. Among the strongest linked variants is rs10811661, located 125 kb upstream of the CDKN2A and CDKN2B genes (for review, [9]). This polymorphism appears to be associated to type 2 diabetes in almost all the ethnic cohorts studies [10–18] and people carrying the TT genotype of this variant showed impaired insulin release and impaired glucose tolerance [19].

**Figure 2:**
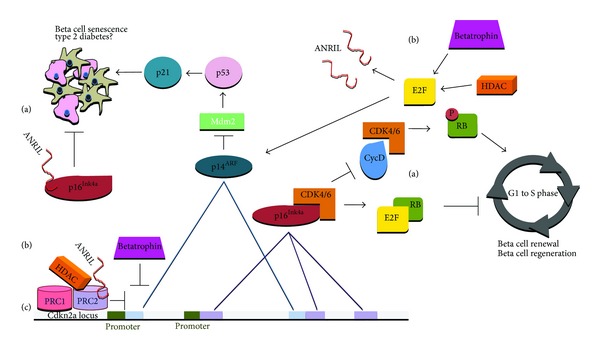
(a) Cell cycle regulation by p16^Ink4b^/p14^ARF^. p16^Ink4b^ inhibits CDK4/6 activity, preventing RB phosphorylation, thus preventing G1/S transition. Inactivation of CDK4/6 promotes Rb/E2F1 association down-regulating transcription of E2F1 target genes, which would trigger G1/S transition. p14^ARF^ inhibits MDM2 activity thereby stabilizing p53 and promoting beta cell senescence. (b) Regulation of beta cell Cdkn2a locus. Binding of PRC1 and PRC2 complex proteins to the p16/p14ARF promoter results in formation of heterochromatin leading to suppression of transcription. HDACs also repress Cdkn2a locus but they conform a regulatory feedback loop: release of E2F1 which up-regulates p14^ARF^ leading to an inhibition of Mdm2 and inducing senescence through p21-p53 pathway. Betatrophin promotes beta cell proliferation by inhibiting Cdkn2a and increasing expression levels of cyclins and E2F1 (through a still unknown mechanism). lncRNA ANRIL, transcribed from the Cdkn2a locus, directly binds to the p16^Ink4b^ transcript and also recruits the PRC complexes to repress the transcription of genes at this locus. ANRIL is induced by E2F1 after DNA damage. (c) Cdkn2a locus showing exons of p16^Ink4b^/p14^ARF^ genes involved in alternate splicing. Promoter regions are shown in green.

The ARF/INK4A (Cdkn2a) locus spans around 35 kilobases on human chromosome 9p21.25 that includes the linked tumor suppressor genes p16^INK4a^ and p14^ARF^ (p19^ARF^ in mice) that trigger the anti-proliferative activities of both RB and p53. While p14^ARF^ is transcribed from exon 1b and exon 2, p16^Ink4A^ is transcribed from exon 1a localized 20 kb downstream of 1b and exons 2 and 3. As described in more detail below and shown in Figure 2, p16^Ink4a^ binds to CDK4/6 inhibiting its kinase activity thereby preventing Rb phosphorylation (pRb), while p14^Arf^ inhibits the ubiquitin ligase activity of MDM2, thereby stabilizing p53. Rb remains associated with transcription factor E2F1 localizing it to the cytoplasm and thus preventing transcription of E2F1 target genes that, as mentioned above, are crucial for the G1/S transition [20].

Beta cells express most of the known cell cycle inhibitors, including p16^INK4a^, p18^INK4c^, p21^CIP1^, p27^Kip1^, p53, and Rb. In contrast, and according to the negative regulation model explained in the introduction, there is much less redundancy of cell cycle activators in the beta cell. This model converges in mouse and human models. For example, rodent beta cells express only CDK4 and not CDK6, whereas most other cell types express both of these proteins. Mouse beta cells express all three D cyclins, D1, D2, D3, but the mRNA expression of D2 is significantly higher than both D1 and D3 with only D2 detectable by immunohistochemistry [2]. In order to establish another regulation point of this pathway in beta cells, Fiaschi-Taesch and colleagues [21, 22] have delineated the repertoire of G1/S regulatory proteins present in the adult human islet and have used this information to develop what they call the “human islet G1/S proteome”. These studies state that although the G1/S molecules are mainly considered to be nuclear proteins, they are present principally in the cytoplasm, where possibly they would not be able to regulate cell cycle progression. Furthermore, the only nuclear G1/S molecules are the cell cycle inhibitors, pRb, p57, and p21. p16^Ink4a^ remains in the nucleus in only 8.4% of beta cells under basal conditions. Which in turns becomes nuclear in 16.1% under induction of proliferation. Cell cycle activators as cyclins or Cdks, necessary to drive *β*-cell proliferation are present in the cytoplasm, not in the nuclear compartment.

Regarding the role of E2F in beta cells, it is well known that the increased expression of E2F contributes to the uncontrolled proliferation of cancer cells, but there is increasing evidence for a Cdk4–E2F1–pRB-specific role in metabolism. To that extent, there is the finding that some specific polymorphisms in the Cdk4 gene could contribute to type 2 diabetes-associated obesity [23]. In that sense, studies E2F1^−/−^ mice show impaired postnatal pancreatic growth that triggers a reduction in pancreatic size with the subsequent impaired glucose homeostasis [24]. Moreover, the CDK4-pRB-E2F1 pathway is activated by glucose through the insulin pathway in beta cells, leading to increased Kir6.2 expression that induces insulin secretion [25–27].

## 3. ARF/INK4A and Beta Cell Senescence

Emerging evidence indicates that proliferation of pancreatic beta cells is an important mechanism not only to maintain homeostasis in the endocrine pancreas but also for adapting islet function to changes in metabolic demands [9, 28, 29]. The inability of the beta cells to expand and compensate for the changing insulin demand can contribute to the pathogenesis of diabetes. Several studies suggest that beta cell proliferation declines with age [30, 31] and this age-dependent decline in the beta cell proliferation could curtail the ability of the endocrine pancreas to respond to metabolic changes. Furthermore, the cell-intrinsic genetic and epigenetic mechanisms regulating the age-dependent decline of beta cell proliferation [32].

On their review, Gunasekaran and cols compile contradictory studies on the age-related effects on the beta cell. While some of them found that insulin sensitivity decreases with age, others shown that plasma glucose clearance was found to be dependent on the waist-to-hip ratio and not age, with the exception of older people with pre-existing impaired glucose tolerance or type 2 diabetes. Even if these studies come into contradiction, there is evidence that with age, beta cells show decreased expression of cell cycle activators with simultaneous increases in expression of cell cycle inhibitors. Controversial findings and opinions regarding the balance of cell cycle inhibitors and activators have been found. Gunasekaran's compendium describes several studies showing that loss of a single cell cycle inhibitor does not accelerate beta cell cycle progression, whereas loss of multiple inhibitors enhances beta cell proliferation [2]. In the opposite sense, other groups describe that p16^Ink4a^ and p19^Arf^ expression (mRNA) was increased significantly with aging in pancreatic islets, but not other Cdk inhibitors examined, including p15, p18, p21, p27. This up regulation has been linked to reduction in the proliferative capacity of aged beta cells [32, 33].

Aging is associated with replicative senescence and p16^Ink4a^ levels increase with aging in most mammalian tissues. The levels of p16^Ink4a^ in an individual can be predicted by stochastic model that takes into consideration the subjects' age. According to this model, p16^Ink4a^ levels exponentially increase with age in a p16^Ink4a^-dependent manner and reach a plateau.

Increased p16^Ink4a^ levels in the pancreas during aging (independent of telomere shortening) inhibit the proliferation of beta cells and decrease their ability to respond to injury. While the beta cells of the p16^Ink4a^ knockout mice were able to proliferate in response to injury, beta cells with ectopic expression of p16^Ink4a^ showed reduced proliferative response confirming the association between p16^Ink4a^ and beta cell senescence [20]. In addition, overexpression of p16^Ink4a^ in transgenic mice caused a reduction of islet proliferation in younger more than in older animals.

Expression of p16^Ink4a^ and p14^ARF^ are regulated by promoter hypermethylation through proteins of the PRC1 and PRC2 complexes of the Polycomb group (PcG) of transcriptional repressor proteins. MLL1 and PcG directly control the Ink4a/Arf locus through chromatin epigenetic modifications and the loss of these repressive epigenetic marks leads to a shift of the replication timing of the locus, both in senescent and Polycomb mutant cells [34].

Bmi-1 is a transcription factor, member of the PcG repression complex 1 (PRC1) that inhibits senescence by inhibiting transcription of p16^Ink4a^. Thus Bmi-1 behaves as an oncogene and is a marker of tumor stem cells. Ezh2 (Enhancer of zeste homolog 2) belongs to the PRC2 complex, it is a histone methyltransferase which represses Ink4a/Arf in islet beta cells, with activity specific for histone H3 K27 [35, 36]. The ability of Bmi-1 to decrease transcription from the Ink4a locus depends on the presence of Ezh2 and other components of the PRC2 complex.

Kotake et al. [37] showed that the removal of pRb from cells resulted in the loss of histone H3 K27 trimethylation leading to the loss of Bmi-1 recruitment to the Ink4a/Arf locus. Moreover, pRb is also shown to be necessary for Bmi-1 function in the transcription repression of Ink4a/Arf. There is a feedback loop between p16^Ink4a^ and Rb: phosphorylation which results in increased p16^Ink4a^ expression and inhibition of CDK4/6 [20]. Other regulatory feedback loop of the locus relays on histone deacetylases (HDAC), p53 is required for both HDAC and PcG to repress p14^Arf^ expression [38], at the same time HDAC1 is involved in the release of E2F1 which in turn could up-regulate p14^Arf^, leading to an inhibition of Mdm2 activity, the subsequent activation of p53 and induction of senescence through p21 [20, 39]. Previously described by Wang and colleagues [40], HBP1 induces premature senescence through upregulating p16^Ink4A^ expression in primary cells by targeting the p16^Ink4A^ promoter, by interacting and recruiting p300/CBP, whereas HDAC4 represses HBP1-induced p16^Ink4a^ expression, thus represses HBP1-induced premature senescence.

## 4. New Insights into INK4a/ARF Regulation of Beta Cell Proliferation

The factors explained below are well known to have a specific role in regulation of beta cell proliferation under physiological and pathological conditions. However, the molecular pathways in which they are involved are not fully studied, but they could be promising targets for expanding functional pancreatic islets in diabetes.

## 5. lncRNAs

Long non-coding RNAs (lncRNAs) are a new class of regulatory RNAs that are defined as transcribed RNA molecules ranging in length from 200 to 100,000 nucleotides and lacking protein-coding capacity. Islet lncRNAs show a marked cell-type specific expression pattern, Morán and colleagues [41] have integrated transcriptional and chromatin maps to systematically annotate lncRNA genes in human pancreatic islet cells and they state that some lncRNAs are dysregulated in type 2 diabetes or map to susceptibility loci. Furthermore, orthologous transcripts in mice are dynamically regulated in a similar manner as human islet lncRNAs. In the case of Cdkn2a, a long non-coding RNA, ANRIL (antisense non-coding RNA), also transcribed from the locus, is involved in the epigenetic regulation of the Cdkn2a locus by direct binding to the p16^Ink4b^ transcript and recruiting the PRC complexes to repress the transcription of genes at this locus. ANRIL is targeted by PRC2 to the Ink4a/Arf/Ink4b locus [42]. Another regulation point regarding ANRIL and cell cycle inhibition is that ANRIL is induced by E2F1 transcription factor after DNA damage, and thus the elevated ANRIL levels suppress the expression of p14^Arf^ and p16^Ink4a^ at the late-stage of DNA damage response (DDR), forming a negative feedback loop to the DDR [43]. Recent studies showed that single nucleotide polymorphisms mapped in the ANRIL as well as in Cdkn2a locus sequences are linked to several pathologic conditions, including type 2 diabetes [39].

## 6. Betatrophin

Yi et al. [44] have described a peptide whose overexpression in mouse liver produces a secreted protein that significantly and specifically promotes pancreatic beta cell proliferation and beta cell mass expansion and, consequently, improves glucose tolerance. Hence, this peptide has been called betatrophin. Expression levels of cyclins (cyclins A1, A2, B1, B2, D1, D2, and F), CDKs (CDK1 and CDK2), and E2Fs (E2F1 and E2F2) increase, whereas cell cycle inhibitors (Cdkn1a and Cdkn2a) decrease in islets of betatrophin-injected mice compared to control-injected mice. The mechanism of action for betatrophin still remains unknown, if it acts directly or not on the beta cells for controlling their proliferation. Betatrophin is not a novel protein, it is also known as ANGPTL8, TD26, RIFL and Lipasin. Previously to the study of Yi and colleagues, it has been shown that betatrophin is expressed at the highest levels in liver and adipose tissue, and is up-regulated by feeding and suppressed by fasting [45, 46]. To date the betatrophin receptor has not been identified, and maybe other cofactors are acting in the specificity of the betatrophin effect on beta cell mass. However, promising opportunities are open with regard to betatrophin and beta cell mass regeneration.

## 7. Conclusions

Type 1 and 2 diabetes can be reversed by replacement of beta cell mass, as demonstrated by pancreas and islet transplantation [47]. However, it has limited applicability, given the shortage of organ donors and the need for chronic immunosuppression. Regeneration of beta cell mass is one promising approach to replace the deficit in beta cell mass in diabetic patients [32]. The results stated in this review support that modulation of the Ink4a/Arf locus plays a critical role in regulating pancreatic beta cell proliferation during aging and regeneration. A strategy of beta cell proliferation improvement based in Ink4a/Arf genes inhibition could help to develop new regeneration approaches. One of the main concerns about inhibiting the action of these genes is that they are anti-oncogenes, and could trigger tumour development [48]. To date, few studies show specific inhibitors of p16 and/or p19 genes. Recently, the direct derepression of p21 and p16^Ink4a^ caused by loss of AP4 gene (a c-Myc transcription factor) in fibroblasts has been shown to be sufficient to mediate cellular senescence [49]. In beta cells, a recent and promising study shows that a bioavailable HNF4*α* (a nuclear receptor transcription factor) antagonist induced *β*-cell replication in rabbits and mice. Moreover, this compound promotes alpha, beta and delta cell replication in beta cell ablated mice, and repressed the expression of multiple cyclin-dependent kinase inhibitors, including p16^Ink4a^ [50]. Understanding the regulation of Ink4a/Arf locus could reveal the molecular basis of reduced beta cell proliferation with aging and also be extremely useful in devising strategies for beta cell regeneration.
